# Exercise capacity in patients with psoriasis assessed by cardiopulmonary exercise testing: a case-control study^؆^

**DOI:** 10.1016/j.abd.2026.501470

**Published:** 2026-10-31

**Authors:** Hanife Baykal Şahin, Arzu Ferhatosmanoğlu, Leyla Baykal Selçuk

**Affiliations:** a Department of Physical Medicine and Rehabilitation, Faculty of Medicine, Karadeniz Technical University, Trabzon, Turkey; b Department of Dermatology, Faculty of Medicine, Karadeniz Technical University, Trabzon, Turkey

**Keywords:** Cardiopulmonary exercise test, Cardiorespiratory fitness, Exercise tolerance, Psoriasis

## Abstract

**Background:**

Psoriasis is a systemic inflammatory disease associated with cardiovascular and metabolic comorbidities. Although reduced physical activity is frequently reported in these patients, objective data on physiological exercise capacity remains limited.

**Objective:**

To evaluate exercise capacity in patients with psoriasis using Cardiopulmonary Exercise Testing (CPET), the gold standard method, and compare the results with those of healthy controls.

**Methods:**

This cross-sectional study included 32 patients with psoriasis (off systemic therapy for ≥6-months) and 33 age- and sex-matched healthy controls. Participants underwent symptom-limited CPET on a cycle ergometer. Disease severity was assessed using the Psoriasis Area and Severity Index (PASI), and Physician Global Assessment (PGA).

**Results:**

Patients with psoriasis demonstrated significantly lower exercise capacity compared to controls. Peak VO₂, percentage of predicted peak VO₂, VO₂ at AT, and work rate were all significantly lower (all p < 0.01). Peak VO₂ showed significant negative correlations with BMI, PASI, and PGA (all p ≤ 0.001). In multiple linear regression analysis, age (β = -0.17, p = 0.017), BMI (β = -0.30, p = 0.007), and PASI score (β = -0.47, p = 0.001) were identified as independent predictors of peak VO₂, explaining 63.4% of its variance (R² = 0.634, p < 0.001).

**Study limitations:**

The cross-sectional design and relatively small single-center sample limit causal inference.

**Conclusions:**

Patients with psoriasis demonstrated reduced exercise capacity compared with healthy controls, and disease severity was independently associated with lower peak VO₂. However, due to the cross-sectional design and the potential influence of unmeasured cardiometabolic and lifestyle-related confounders, causality cannot be established, and further prospective studies are required.

## Introduction

Psoriasis is a chronic immune-mediated inflammatory disease characterized by erythematous, scaly plaques primarily affecting the skin and sometimes the joints.^1^ Disease is increasingly recognized as a systemic disease associated with multiple comorbidities, including cardiovascular disease, obesity, metabolic syndrome, and depression. These comorbid conditions contribute significantly to the overall disease burden and negatively impact long-term health outcomes in patients with psoriasis.^1^, ^2^, ^3^

In recent years, growing evidence has suggested that lifestyle factors play an important role in the pathophysiology and clinical course of psoriasis. Physical inactivity is particularly important because it contributes to cardiovascular risk, metabolic dysfunction, and chronic systemic inflammation, which are frequently observed in patients with psoriasis.^4^, ^5^ Patients with psoriasis may also experience disease-specific barriers to physical activity, including skin discomfort, embarrassment related to visible lesions, and psychosocial limitations, which can reduce participation in regular exercise and limit access to health-promoting levels of physical activity.^6^

Cardiorespiratory Fitness (CRF), commonly assessed through exercise capacity measurements, reflects the integrated function of the cardiovascular, respiratory, and muscular systems during physical exertion. Higher CRF levels are strongly associated with reduced cardiovascular morbidity, improved metabolic health, and lower all-cause mortality.^7^ Importantly, CRF represents not only habitual physical activity but also physiological capacity to perform sustained aerobic exercise. Therefore, assessment of exercise capacity provides a more objective indicator of physical performance than self-reported physical activity levels.^7^, ^8^

Although the relationship between psoriasis and physical activity has been investigated, the available evidence remains limited and heterogeneous. Questionnaire-based studies have consistently reported lower physical activity levels among patients with psoriasis compared with healthy individuals, although such self-reported measures may be affected by recall bias and may not accurately reflect true exercise capacity.^9^ Objective assessments of exercise performance in psoriasis are relatively scarce. For example, Demirel et al.^10^ evaluated aerobic capacity using a submaximal exercise protocol and found no significant difference in maximal aerobic capacity between patients with mild-to-moderate psoriasis and healthy controls, despite differences in daily physical activity levels. In contrast, studies using performance-based tests such as the six-minute walk test have demonstrated reduced exercise tolerance and impaired physical performance in this population.^11^ Furthermore, emerging epidemiological evidence suggests that reduced CRF may be associated with psoriasis development and disease outcomes. A large prospective cohort study including more than one million Swedish men reported that low CRF in late adolescence was associated with an increased long-term risk of psoriasis and psoriatic arthritis, while population-based analyses have shown that individuals with psoriasis tend to have lower CRF levels than those without the disease, independent of Body Mass Index (BMI) and physical activity levels.^8^, ^12^

Beyond observational findings, increasing physical activity has also been shown to improve clinical and metabolic outcomes in patients with psoriasis. Interventional studies indicate that structured physical activity programs may lead to improvements in psoriasis severity, cardiovascular risk factors, psychological well-being, and functional capacity.^6^ These findings suggest that exercise capacity may play an important role in the clinical management and overall health status of patients with psoriasis.

Despite these observations, objective evaluation of exercise capacity using Cardiopulmonary Exercise Testing (CPET) in patients with psoriasis remains extremely limited. CPET provides comprehensive physiological assessment by measuring parameters such as peak oxygen uptake (VO₂peak), oxygen pulse, anaerobic threshold, and work rate, allowing detailed characterization of cardiopulmonary performance during exercise.^13^ Such measurements may provide valuable insights into the functional limitations associated with psoriasis and its systemic manifestations.

Therefore, the aim of the present study was to evaluate exercise capacity in patients with psoriasis using CPET and to compare these parameters with those of healthy individuals.

## Methods

### Study design and participants

This cross-sectional study included 32 patients with psoriasis and 33 age- and sex-matched healthy controls. Patients were consecutively recruited from the dermatology outpatient clinic between January 2024 and February 2026. The diagnosis of psoriasis was established clinically by experienced dermatologists. Adults aged 18–65 years with clinically confirmed psoriasis were eligible for inclusion. Patients with known psoriatic arthritis, severe obesity preventing adequate CPET performance, active infection, uncontrolled cardiopulmonary disease, or other musculoskeletal or neurological conditions limiting exercise performance were excluded. To minimize the potential confounding effects of systemic therapies on inflammatory status and exercise capacity, only patients who had not received systemic psoriasis treatment, including methotrexate, cyclosporine, biologic agents, or systemic corticosteroids, for at least six months prior to enrollment were included in the study. Healthy controls were recruited from hospital staff and volunteers with no history of psoriasis or other chronic inflammatory diseases. All participants provided written informed consent prior to participation. The study protocol was approved by the Institutional Ethics Committee and was conducted in accordance with the principles of the Declaration of Helsinki.

### Clinical assessment

Demographic and anthropometric characteristics, including age, sex, height, weight, BMI, smoking status, and alcohol consumption, were recorded for all participants. In patients with psoriasis, disease-related characteristics, including disease duration and disease severity, were documented. Disease severity was evaluated using the Psoriasis Area and Severity Index (PASI), a widely used tool that assesses erythema, induration, scaling, and the extent of body surface area involvement, and the Physician Global Assessment (PGA).^14^ Dermatology-specific quality of life was assessed using the Dermatology Life Quality Index (DLQI), a validated questionnaire widely used in patients with psoriasis.^15^ It consists of 10 questions assessing the impact of skin disease on different aspects of daily life, with total scores ranging from 0 to 30, with higher scores indicating greater impairment in dermatology-related quality of life.

Physical activity levels were assessed using the International Physical Activity Questionnaire (IPAQ), a self-reported instrument that estimates the frequency and duration of walking, moderate-intensity, and vigorous-intensity physical activities over the previous seven days and expresses total physical activity as Metabolic Equivalent Task minutes per week (MET-min/week).^16^ Psychological status was assessed using the Hospital Anxiety and Depression Scale (HADS). It is a 14-item questionnaire consisting of Anxiety (HADS-A) and Depression (HADS-D) subscales, each comprising 7-items scored on a 4-point Likert scale (0–3), with subscale scores ranging from 0 to 21 and scores ≥ 8 indicating clinically significant symptoms.^17^

### Cardiopulmonary exercise testing

CPET was performed using an electronically braked cycle ergometer with a ramp protocol. Following a short resting period and a brief warm-up phase, the workload was progressively increased in a continuous ramp manner until symptom-limited maximal exercise was achieved. A Respiratory Exchange Ratio (RER) ≥ 1.10 was considered indicative of maximal effort. Respiratory gas exchange was measured breath-by-breath throughout the test using a metabolic cart. Standard electrocardiographic monitoring and blood pressure measurements were performed during exercise.

Peak oxygen uptake (VO₂peak), expressed in ml/kg/min and as a percentage of predicted values, was recorded as the highest value obtained during the test. Oxygen uptake at the Anaerobic Threshold (VO₂ at AT) was determined using the V-slope method. Additional parameters including Work Rate (WR), oxygen pulse, maximal Heart Rate (HRmax), and Ventilatory Efficiency (VE/VCO₂ slope) were also recorded.

### Statistical analysis

Statistical analyses were performed using SPSS software (version 26; IBM Corp., Armonk, NY, USA). Continuous variables were expressed as mean ± Standard Deviation (SD), while categorical variables were presented as numbers and percentages. Normality of continuous variables was assessed using the Shapiro-Wilk test. Comparisons between the psoriasis and control groups were performed using the independent samples t-test for continuous variables and the Chi-Square test for categorical variables. Correlations between clinical variables and CPET parameters in patients with psoriasis were evaluated using Spearman correlation analysis. To identify independent predictors of exercise capacity, multiple linear regression analysis was performed with peak VO₂ as the dependent variable. Variables associated with peak VO₂ in univariate analyses were entered into the multivariable model. A p-value < 0.05 was considered statistically significant.

A post hoc power analysis based on the observed between-group difference in peak VO₂ demonstrated that the study achieved 93% statistical power at a two-sided α level of 0.05.

## Results

A total of 65 participants were included in the study, comprising 32 patients with psoriasis and 33 healthy controls. The demographic and clinical characteristics of the study population are summarized in Table 1. There were no significant differences between the two groups in terms of age, sex distribution, anthropometric measurements, smoking status, or alcohol consumption (all p > 0.05). In patients with psoriasis, the mean disease duration was 11.6 ± 9.8 years, with a mean PASI score of 10.6 ± 4.0 and a mean PGA score of 4.72 ± 1.35. The mean DLQI score was 11.3 ± 6.9.

**Table 1:** Demographic and clinical characteristics.

	Psoriasis (n = 32)	Control (n = 33)	p-value
**Age (years), mean ± SD**	42.6 ± 9.5	42.4 ± 7.6	0.926
**Female sex, n (%)**	17 (53%)	20 (61%)	0.72
**Height (cm)**	167.0 ± 7.0	165.0 ± 10.1	0.342
**Weight (kg)**	81.6 ± 16.0	80.2 ± 14.5	0.711
**BMI (kg/m²)**	29.1 ± 4.7	29.4 ± 4.7	0.792
**Smoking, n (%)**	20 (63%)	16 (48%)	0.38
**Alcohol, n (%)**	4 (12%)	5 (15%)	0.967
**Disease duration (years), mean ± SD**	11.6 ± 9.8		
**PASI score, mean ± SD**	10.6 ± 4.0		
**PGA score, mean ± SD**	4.72 ± 1.35		
**DLQI score, mean ± SD**	11.3 ± 6.9		
**IPAQ score**	687.5 ± 858.0	1627.6 ± 1204.0	0.0006
**HADS-Depression**	8.94 ± 3.77	4.39 ± 2.18	<0.001
**HADS-Anxiety**	8.81 ± 4.61	5.67 ± 2.77	0.0017

SD, Standard Deviation; BMI, Body Mass Index; PASI, Psoriasis Area and Severity Index; PGA, Physician Global Assessment; DLQI, Dermatology Life Quality Index; IPAQ, International Physical Activity Questionnaire; HADS, Hospital Anxiety and Depression Scale.

Physical activity levels were significantly lower in the psoriasis group compared with the control group. The mean IPAQ score was 687.5 ± 858.0 in patients with psoriasis and 1627.6 ± 1204.0 in healthy controls (p = 0.0006). In addition, patients with psoriasis exhibited significantly higher levels of both depression and anxiety as assessed by the HADS compared with controls (p < 0.01 for both comparisons).

CPET results are presented in Table 2. Patients with psoriasis demonstrated significantly lower exercise capacity compared with healthy controls. Peak VO₂ and the percentage of predicted peak VO₂ were significantly reduced in the psoriasis group (p = 0.0009 and p < 0.001, respectively). Similarly, VO₂ at AT, work rate, oxygen pulse, and maximal heart rate were significantly lower in patients with psoriasis (all p < 0.01). In contrast, ventilatory efficiency assessed by the VE/VCO₂ slope did not differ significantly between the groups (p = 0.227). The comparison of peak VO₂ values between the two groups is illustrated in Fig. 1.

**Table 2:** Cardiopulmonary exercise test results.

	Psoriasis (n = 32) (mean ± SD)	Control (n = 33) (mean ± SD)	p-value
**Peak VO₂ (mL/kg/min)**	15.70 ± 3.89	19.94 ± 5.71	0.0009
**Peak VO₂ (%)**	59.6 ± 15.3	74.9 ± 9.1	<0.001
**VO₂ at Anaerobic Treshold (%)**	66.6 ± 12.3	76.5 ± 14.8	0.0047
**Work Rate (WR)**	74.6 ± 23.1	104.6 ± 32.9	<0.001
**O₂ pulse**	9.41 ± 2.61	11.73 ± 3.64	0.0045
**HRmax**	134.8 ± 14.6	147.0 ± 18.4	0.0045
**VE/VCO₂ slope**	28.75 ± 3.35	27.66 ± 3.86	0.227

**Figure 1:**
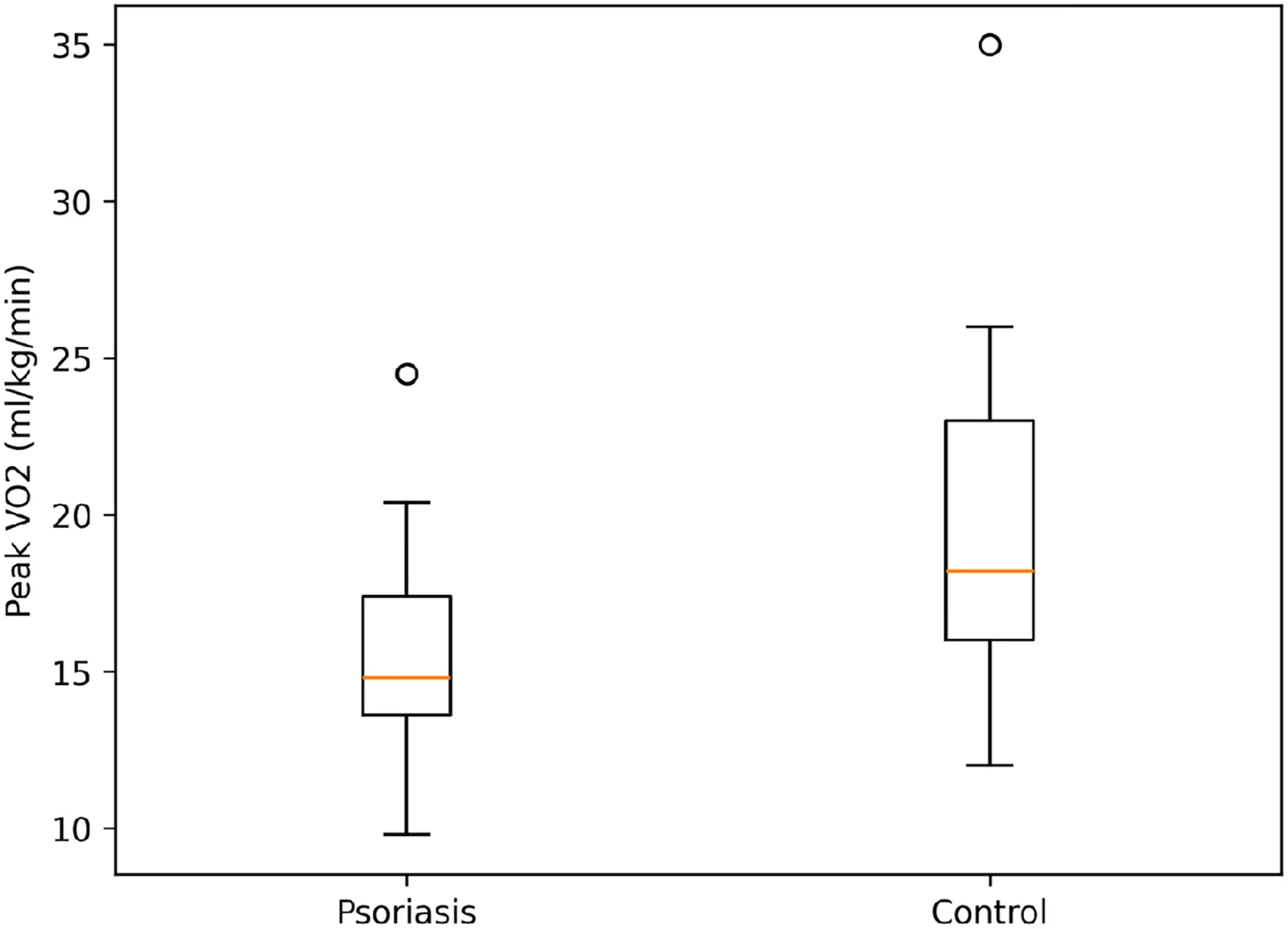
Comparison of peak oxygen uptake (VO₂peak) between patients with psoriasis and healthy controls.

The correlations between clinical characteristics and CPET parameters in patients with psoriasis are presented in Table 3. Peak VO₂ showed a significant negative correlation with BMI (*r* = -0.55, p = 0.001), PASI score (*r* = -0.65, p < 0.001), PGA score (r = -0.65, p < 0.001), and DLQI score (*r* = -0.57, p < 0.001). Similarly, the percentage of predicted peak VO₂ was negatively correlated with PASI (*r* = -0.52, p = 0.002) and PGA (*r* = -0.63, p < 0.001). Work rate was significantly negatively correlated with PASI (*r* = 0.46, p = 0.008), PGA (*r* = -0.51, p = 0.003), and DLQI (*r* = -0.68, p < 0.001). Disease duration was not significantly correlated with peak VO₂ or most CPET parameters.

**Table 3:** Spearman correlations between clinical characteristics and cardiopulmonary exercise test parameters in patients with psoriasis.

	Peak VO₂ (mL/kg/min)	Peak VO₂ (%)	VO₂ at AT (%)	Work Rate	O₂ Pulse	HRmax	VE/VCO₂ slope
**Age**	-0.31 (0.085)	-0.02 (0.93)	-0.02 (0.91)	-0.31 (0.087)	-0.26 (0.143)	-0.31 (0.086)	-0.07 (0.690)
**BMI**	-0.55 (0.001)	-0.25 (0.175)	-0.13 (0.463)	-0.15 (0.404)	0.45 (0.010)	-0.48 (0.005)	-0.44 (0.011)
**Disease duration**	-0.06 (0.754)	-0.06 (0.728)	0.39 (0.025)	-0.11 (0.565)	-0.14 (0.459)	-0.22 (0.227)	-0.06 (0.739)
**PASI**	-0.65 (<0.001)	-0.52 (0.002)	0.31 (0.089)	-0.46 (0.008)	-0.30 (0.092)	-0.13 (0.477)	0.16 (0.387)
**PGA**	-0.65 (<0.001)	-0.63 (<0.001)	0.10 (0.573)	-0.51 (0.003)	-0.02 (0.906)	-0.31 (0.083)	0.08 (0.661)
**DLQI**	-0.57 (<0.001)	-0.45 (0.010)	0.42 (0.017)	-0.68 (<0.001)	-0.42 (0.016)	-0.30 (0.098)	0.03 (0.866)
**IPAQ**	-0.20 (0.279)	0.15 (0.419)	-0.16 (0.377)	-0.35 (0.052)	-0.52 (0.002)	0.06 (0.763)	0.08 (0.656)

*r* (p) values are given.

BMI, Body Mass Index; PASI, Psoriasis Area and Severity Index; PGA, Physician Global Assessment; DLQI, Dermatology Life Quality Index; IPAQ, International Physical Activity Questionnaire; HADS, Hospital Anxiety and Depression Scale.

To identify independent predictors of exercise capacity in patients with psoriasis, a multiple linear regression analysis was performed with peak VO₂ as the dependent variable (Table 4). Age (β = -0.17, p = 0.017), BMI (β = -0.30, p = 0.007), and PASI score (β = -0.47, p = 0.001) were identified as independent predictors of peak VO₂. In contrast, the IPAQ score was not significantly associated with peak VO₂ (p = 0.841). The overall regression model explained 63.4% of the variance in peak VO₂ (R² = 0.634, adjusted R² = 0.579, p < 0.001).

**Table 4:** Multiple linear regression analysis identifying predictors of peak VO₂ in patients with psoriasis.

Variable	Coefficient (β) (Std. Error)	p-value	95% CI
Age	-0.17 (0.07)	0.017	-0.315 – -0.034
BMI	-0.30 (0.10)	0.007	-0.508 – -0.090
PASI	-0.47 (0.12)	0.001	-0.725 – -0.220
IPAQ score	-0.0002 (0.001)	0.841	-0.002 – 0.001

Model statistics: R² = 0.634, Adjusted R² = 0.579, F = 11.68, p < 0.001.

Dependent variable: Peak VO₂ (mL/kg/min).

BMI, Body Mass Index; PASI, Psoriasis Area and Severity Index; IPAQ, International Physical Activity Questionnaire.

## Discussion

The present study evaluated exercise capacity using CPET in patients with psoriasis and compared CPET-derived parameters with those of healthy controls. The main findings indicate that patients with psoriasis have significantly reduced exercise capacity across multiple objective measures, including peak VO₂, VO₂ at the AT, work rate, oxygen pulse, and maximal heart rate. Furthermore, disease severity as assessed by PASI and PGA scores, BMI, and age emerged as independent predictors of peak VO₂, collectively accounting for approximately 63% of the variance in exercise capacity within the psoriasis group.

To our knowledge, this is one of the few studies to employ CPET for the objective assessment of exercise capacity in patients with psoriasis. Previous work in this area has been largely limited to self-reported physical activity questionnaires and performance-based field tests. Questionnaire-based investigations have consistently demonstrated lower physical activity levels among patients with psoriasis compared with healthy individuals.^9^ However, the use of objective CPET in the present study adds an important dimension that self-reported measures cannot provide: a direct, physiologically grounded quantification of cardiorespiratory reserve. Although patients with psoriasis demonstrated significantly lower IPAQ scores than controls, self-reported physical activity was not independently associated with peak VO₂ in multivariable analysis. This finding suggests that the impaired exercise capacity observed in psoriasis may not be explained solely by reduced habitual physical activity and may additionally reflect systemic physiological effects of the disease itself. The substantially lower peak VO₂ values observed in psoriasis patients, alongside reduced VO₂ at AT, oxygen pulse, and work rate, demonstrate that exercise limitation in this population extends beyond self-reported physical inactivity and reflects measurable impairment of integrated cardiopulmonary and muscular function. The observation that VE/VCO₂ slope did not differ significantly between groups is noteworthy: it suggests that ventilatory efficiency is relatively preserved in psoriasis patients, and that the observed exercise limitation is unlikely to be driven primarily by pulmonary ventilatory dysfunction.^18^ Rather, the reductions in peak VO₂, oxygen pulse, and work rate point toward peripheral and cardiovascular determinants of exercise impairment.

Similar impairments in CRF have also been reported in other chronic inflammatory diseases, particularly psoriatic arthritis. Recent studies using CPET demonstrated reduced VO₂peak and impaired CRF in patients with psoriatic arthritis, with disease activity and adiposity independently associated with lower exercise capacity.^19^ These findings support the concept that chronic systemic inflammation may adversely affect aerobic capacity across immune-mediated inflammatory disorders. Comparable reductions in physical activity and exercise tolerance have also been described in rheumatoid arthritis and systemic lupus erythematosus, where inflammation, pain, fatigue, and cardiometabolic comorbidities collectively contribute to functional impairment.^20^, ^21^

A key finding of the present study was the strong and significant negative correlation between disease severity and exercise capacity. Both PASI and PGA scores showed robust negative correlations with peak VO₂, percentage of predicted peak VO₂, and work rate. These relationships persisted in multiple linear regression analysis. PASI remained significantly associated with peak VO₂ after adjustment for age and BMI. These findings suggest an association between greater psoriasis severity and reduced CRF; however, causal relationships cannot be established due to the cross-sectional study design. Chronic low-grade systemic inflammation, a hallmark of psoriasis that intensifies with increasing disease activity, has been shown to impair endothelial function, alter skeletal muscle metabolism, and affect cardiovascular performance, thereby contributing to reduced aerobic exercise capacity.^1^, ^2^, ^22^ The present findings support the view that psoriasis should not be regarded solely as a skin condition, but as a systemic disease with broad functional consequences.

Quality of life, as measured by the DLQI, was also significantly negatively correlated with peak VO₂ and work rate. This association may reflect a bidirectional relationship: reduced exercise capacity may contribute to poorer physical functioning and psychological well-being, while the psychological burden and social stigma associated with visible psoriatic lesions, reflected in higher DLQI scores, may itself act as a barrier to physical activity and structured exercise participation.^23^ Consistently, patients with psoriasis in the present study demonstrated significantly higher levels of anxiety and depression compared with controls, as assessed by the HADS. These psychosocial factors, which are well-recognized determinants of physical activity participation, may compound the direct physiological effects of systemic inflammation on exercise capacity, thereby creating a self-reinforcing cycle of physical inactivity, physical deconditioning, and increasing disease burden.^24^, ^25^ Therefore, the observed association between disease severity and exercise capacity should not be interpreted as strictly causal.

The clinical implications of these findings are particularly relevant for the multidisciplinary management of psoriasis. Objective assessment of exercise capacity through CPET may help identify patients at increased cardiovascular and metabolic risk who could benefit from targeted exercise interventions. Structured physical activity programs have been shown to improve psoriasis severity, cardiometabolic risk profiles, psychological wellbeing, and functional capacity in this population.^6^ The present findings suggest that improvements in disease control, alongside exercise training, could yield complementary benefits on CRF. Dermatologists and treating clinicians should consider the broader systemic and functional consequences of active psoriatic disease when formulating management strategies, and integration of physical activity counseling into routine psoriasis care may therefore be warranted.^26^

Several limitations of the present study should be acknowledged. The cross-sectional design precludes causal inference, and the relatively modest sample size may limit statistical power for subgroup analyses. The study population was recruited from a single dermatology outpatient clinic, potentially limiting generalizability to the broader psoriasis population. Dietary habits and smoking exposure were not systematically controlled for and may have influenced exercise capacity. Ethnicity/race was also not systematically recorded, which may limit the generalizability of CPET findings across different populations. Restricting inclusion to patients without recent systemic therapy may have introduced selection bias and may limit the generalizability of the findings to patients receiving biologic or intensive systemic treatment. Hypertension, diabetes mellitus, dyslipidemia, and systemic inflammatory biomarkers were not systematically evaluated and may have acted as residual confounders affecting exercise capacity. The absence of imaging-based assessment of cardiovascular structure and function represents an additional limitation, as subclinical cardiac involvement in psoriasis may contribute independently to exercise capacity impairment.

## Conclusions

The present study demonstrates that patients with psoriasis exhibit significantly reduced CRF as objectively assessed by CPET, with disease severity, BMI, and age emerging as independent predictors of exercise capacity. These findings suggest that psoriasis severity is associated with impaired CRF and support the integration of objective exercise assessment into multidisciplinary clinical management. Moreover, the present results emphasize that psoriasis should be considered not only a dermatologic condition but also a systemic disease with measurable functional consequences affecting cardiopulmonary performance. Future longitudinal studies comparing treated and untreated patients and evaluating changes in CPET parameters following systemic therapy may help clarify the relationship between psoriasis severity and exercise capacity.

## ORCID ID

Arzu Ferhatosmanoğlu: 0000-0002-1784-1645

Leyla Baykal Selçuk: 0000-0001-7956-4033

## Research data availability

The entire dataset supporting the results of this study was published in this article.

## Financial support

None declared.

## Authors’ contributions

Hanife Baykal Şahin: Approval of the final version of the manuscript; data collection, analysis and interpretation; preparation and writing of the manuscript; study conception and planning.

Leyla Baykal Selçuk: Approval of the final version of the manuscript; data collection, analysis and interpretation; manuscript critical review; statistical analysis.

Arzu Ferhatosmanoğlu: Approval of the final version of the manuscript; critical literature review; data collection, analysis and interpretation.

## Ethics approval and consent to participate

This study was approved by the Ethics Committee of Karadeniz Technical University, Faculty of Medicine (Protocol Number: 2024-196). All participants provided written informed consent prior to participation. All procedures were conducted in accordance with the principles of the Declaration of Helsinki.

## Conflicts of interest

None declared.
